# Translation and validation of Greek version of the Pandemic Grief Scale

**DOI:** 10.1192/j.eurpsy.2023.1015

**Published:** 2023-07-19

**Authors:** K. S. Kitsou, M. Bakola, C. Kalogirou, S. Aggelakou-Vaitsi, N. Vaitsis, K. Argyropoulos, M. Kampouraki, E. Gkatsi, K. Tsolaki, M. Vakas, A. Theochari, K. Mavridou, M. Siali, S. Karatzeni, M. Chalkidou, V. Karagianni, N. Kioses, P. Gourzis, E. Jelastopulu

**Affiliations:** 1 Department of Public Health; 2 Department of Psychiatry, University of Patras, Medical School, Patras, Greece

## Abstract

**Introduction:**

Those who have lost loved ones to COVID-19 may be considered at risk of complicated grief. A 5-item mental health screening tool called the Pandemic Grief Scale (PGS) was developed to find likely instances of dysfunctional grief during the pandemic.

**Objectives:**

To develop a Greek version of PGS and to explore the validity and reliability among the general population in Greece in order to further use it as clinical mental health screener.

**Methods:**

We conducted a cross-sectional study between January and April 2022, and 342 persons were recruited. The questionnaire included socio-demographic parameters, the PGS, the Brief Resilience Coping Scale to capture tendencies to cope with stress and the Athens Insomnia Scale to assess the insomnia symptoms. Based on experiences over the previous two weeks, each PGS item is scored on a 4-point scale, from 0 (not at all) to 3 (almost every day), with higher rating and a cut-off of 7 indicating dysfunctional grief. Prior to the psychometric validation a linguistic validation and adaptation in Greek was performed.

**Results:**

A total of 342 patients participated in the study, 67.8 % were females and 27.8% were 18-30 years old. Coefficient Validity Ratio (CVR) results showed that 100% (n = 5) of items were acceptable. Value of Cronbach’s alpha was found 0.848. A one-factor model was conducted by Confirmatory Factor Analysis (CFA), giving acceptable global fit indices. The resulting global fit indices [Standardized Root Mean Square Residual (SRMR) = 0.037, Comparative Fit Index (CFI) = 0.952, Tucker–Lewis Index (TLI)= 0.903] showed that the 5 items in one-factor solution proposed by the primary researchers shouldn’t be rejected for the Greek version. The Bartlett Test of Sphericity was 758.08 (p <0.001). The Kaiser-Meyer-Olkin Measure of Sampling Adequacy was 0.826, showing that the data is suitable for factor analysis. The one-factor solution derived in our study consisted of 5 items. The total explained variance was 64.3 %.

**Conclusions:**

The findings of this research support the PGS psychometric validity and reliability. PGS is suitable to be used in healthcare to identify and assist individuals, who are experiencing this type of pandemic-related dysfunctional grief as it is a screening tool that it’s simple to use, access, and understand.

**Disclosure of Interest:**

None Declared

